# A portrait of electronic nicotine delivery systems use in Brazil: findings from the 2019-2023 Vigitel survey

**DOI:** 10.1590/0102-311XEN120925

**Published:** 2026-05-18

**Authors:** João Ferreira Silva, Antônio Gonçalves, Verônica Silva Carneiro

**Affiliations:** 1 Universidade Estadual do Maranhão, Itapecuru Mirim, Brasil.; 2 Departamento de Medicina, Universidade Federal do Maranhão, São Luís, Brasil.; 3 Programa de Saúde Mental, Secretaria Municipal de Educação, Presidente Dutra, Brasil.

**Keywords:** Electronic Nicotine Delivery Systems, Sociodemographic Factors, Health Behavior, Sistemas Eletrônicos de Liberação de Nicotina, Fatores Sociodemográficos, Comportamentos Relacionados com a Saúde, Sistemas Electrónicos de Liberación de Nicotina, Factores Sociodemográficos, Conductas Relacionadas con la Salud

## Abstract

The use of electronic nicotine delivery systems (ENDS) has been increasing globally, raising concerns about their impact on public health, particularly among young adults. In Brazil, despite strong tobacco control policies, data on the prevalence of ENDS use and its associated factors remain limited. This study aimed to analyze sociodemographic and behavioral factors associated with lifetime ENDS use among Brazilian adults from 2019 to 2023. Data from four national surveys conducted in 2019, 2020, 2021, and 2023, including 126,681 participants, were analyzed. Crude and adjusted logistic regression models were applied to estimate odds ratios and 95% confidence intervals to assess associations between sociodemographic characteristics, health behaviors, and lifetime ENDS use. All analyses were weighted to account for the complex sampling design and stratified by survey year. Lifetime ENDS use was significantly higher among young adults (18-24 years), males, individuals living in the Central-West and South regions, and those reporting binge drinking and smoking. Higher educational attainment and prolonged screen time (≥ 3 hours/day) were also positively associated with ENDS use. Lifetime ENDS use in Brazil is influenced by sociodemographic and lifestyle factors, with higher prevalence among young adult males with higher education levels and residents of specific regions. These findings highlight the need for targeted public health strategies aimed at populations at higher risk to curb the growing use of ENDS and prevent potential health consequences.

## Abstract (pt)

O uso de dispositivos eletrônicos para fumar (DEF) tem aumentado globalmente, suscitando preocupações sobre seus impactos na saúde pública, especialmente entre jovens adultos. No Brasil, apesar das políticas consolidadas de controle do tabaco, dados sobre a prevalência e os fatores associados ao uso de DEF ainda são limitados. O objetivo desse estudo foi analisar fatores sociodemográficos e comportamentais associados ao uso de DEF ao longo da vida em adultos brasileiros entre 2019 e 2023. Foram analisados dados de quatro pesquisas nacionais realizadas em 2019, 2020, 2021 e 2023, totalizando 126.681 participantes. Modelos de regressão logística brutos e ajustados foram empregados para estimar razões de chances e intervalos de 95% de confiança, a fim de avaliar associações entre características sociodemográficas, comportamentos de saúde e uso de DEF ao longo da vida. Todas as análises foram ponderadas para considerar o desenho amostral complexo e estratificadas por ano de pesquisa. O uso de DEF ao longo da vida foi significativamente maior entre jovens adultos (18-24 anos), homens, indivíduos residentes no Centro-oeste e Sul do país e aqueles que relataram consumo excessivo de álcool e tabagismo. Maior nível de escolaridade e tempo prolongado de tela (≥ 3 horas/dia) também se associaram positivamente ao uso de DEF. O uso de DEF ao longo da vida no Brasil é influenciado por fatores sociodemográficos e de estilo de vida, com maior prevalência entre jovens adultos do sexo masculino, com níveis mais elevados de escolaridade e residentes em regiões específicas. Esses achados reforçam a necessidade de estratégias direcionadas de saúde pública voltadas a populações de maior risco, buscando conter o uso crescente de DEF e prevenir possíveis consequências à saúde.

## Abstract (es)

El uso de dispositivos electrónicos para fumar (DEF) ha aumentado en todo el mundo, lo que genera preocupación sobre sus efectos en la salud pública, especialmente entre los jóvenes adultos. En Brasil, a pesar de las políticas establecidas de control del tabaco, los datos sobre la prevalencia y los factores asociados al uso de DEF aún son limitados. El objetivo de este estudio fue analizar los factores sociodemográficos y actitudinales asociados al uso de DEF a lo largo de la vida en adultos brasileños entre el 2019 y el 2023. Se analizaron datos de cuatro encuestas nacionales realizadas en 2019, 2020, 2021 y 2023, con un total de 126.681 participantes. Se emplearon modelos de regresión logística crudos y ajustados para estimar los *odds ratios* y los intervalos de 95% de confianza con el fin de evaluar las asociaciones entre las características sociodemográficas, las actitudes de salud y el uso de DEF a lo largo de la vida. Todos los análisis se ponderaron para tener en cuenta el complejo diseño de muestreo y se estratificaron por año de investigación. El uso de DEF a lo largo de la vida fue significativamente mayor entre jóvenes adultos (18 a 24 años), hombres, individuos residentes en el Centro-Oeste y Sur del país, y aquellos que reportaron un consumo excesivo de alcohol y tabaco. Un nivel más alto de educación y un tiempo prolongado frente a la pantalla (≥ 3 horas/día) también se asociaron positivamente con el uso de DEF. El uso de DEF a lo largo de la vida en Brasil está influenciado por factores sociodemográficos y de estilo de vida, con mayor prevalencia entre jóvenes adultos del sexo masculino, con mayores niveles de educación y residentes en regiones específicas. Estos hallazgos refuerzan la necesidad de contar con estrategias de salud pública específicas dirigidas a las poblaciones de mayor riesgo, buscando frenar el creciente uso de DEF y prevenir posibles consecuencias para la salud.

## Introduction

Electronic nicotine delivery systems (ENDS), also known as vapes, are devices that produce nicotine-containing vapor during use ^1^. ENDS include electronic cigarettes, heated tobacco products, and electronic waterpipes ^2^. Although the tobacco industry promotes them as producing a “harmless vapor”, they in fact generate aerosols composed of nicotine, solvents (such as propylene glycol and vegetable glycerin), flavoring agents, and a variety of toxic substances ^2^ ^,^ ^3^. A study have found the presence of heavy metals (including lead, nickel, and cadmium), volatile organic compounds, formaldehyde, acrolein, and other harmful agents ^3^.

Their use has increased sharply worldwide over the past decade, raising public health concerns due to potential effects on smoking initiation, cessation, and overall health outcomes ^4^ ^,^ ^5^. Although initially marketed as a less harmful alternative to traditional cigarettes, growing evidence indicates multiple health risks, particularly among young adults. These include increased risks of cardiovascular and pulmonary diseases, exposure to toxic substances, physical injuries, and potential nicotine dependence ^6^ ^,^ ^7^. These concerns are largely attributed to marketing strategies that undermine current regulations prohibiting the commercialization of these devices ^7^.

A recent systematic review identified key risk factors associated with ENDS use ^8^, although it did not distinguish between past and current use. The findings suggest that ENDS use is commonly linked to both individual and contextual characteristics, including adolescence, male sex, current or former tobacco use, alcohol and drug consumption (including marijuana), sensation-seeking behavior, and greater affinity for technology (technophilia) ^8^. Social and environmental factors also contribute, such as having friends or parents who smoke, attending private schools, and exposure to ENDS and tobacco advertising, particularly via digital media. Moreover, the widespread perception that ENDS are less harmful than conventional tobacco products facilitates their use, underscoring the need for stricter regulation and more accurate dissemination of health information ^8^.

In Brazil, a country recognized for its strong tobacco control policies, the sale, importation, and marketing of ENDS have been prohibited since 2009 under *Resolution n. 46/2009* of the Brazilian Health Regulatory Agency (Anvisa, acronym in Portuguese) ^9^. Recently, *Resolution n. 855/2024* extended this ban to include the production, distribution, storage, and transportation of ENDS throughout the national territory ^2^. Despite these restrictions, the landscape of nicotine use in the country is changing.

Comprehensive national data on ENDS prevalence reveal important trends. According to the 2019 *Brazilian National Survey of Health* (PNS, acronym in Portuguese), adolescents and young adults aged 15-24 years showed the highest prevalence of ENDS use, with 5.41% reporting lifetime use and 2.38% reporting current use ^2^. Data from the *Brazilian National Survey of School Health* (PeNSE, acronym in Portuguese) also indicate high experimenting rates among adolescents aged 13-17: 26.9% for hookah, 16.8% for electronic cigarettes, and 9.3% for other tobacco products ^10^. Similarly, the *Covitel Study* (2022) found that young adults aged 18-24 years had the highest prevalence of experimenting e-cigarettes (19.7%) and hookah (17%) ^11^.

Studies have consistently identified adolescents and young adults, males, individuals with higher levels of education, and residents of specific regions as key correlates of ENDS use ^5^ ^,^ ^8^ ^,^ ^12^. Given Brazil’s unique socioeconomic and cultural context, a localized multi-year analysis is necessary to assess the persistence of these risk factors over time.

This study addresses this gap by analyzing data from large, nationally representative surveys conducted in 2019, 2020, 2021, and 2023. It examines factors associated with lifetime ENDS use among Brazilian adults. By exploring demographic and behavioral variables, the study provides critical insights to inform targeted public health interventions and policies. Understanding these patterns is essential for developing effective tobacco harm reduction strategies and mitigating the potential public health consequences of the growing use of ENDS.

## Methods

### Study design and participants

**Figure 1:**
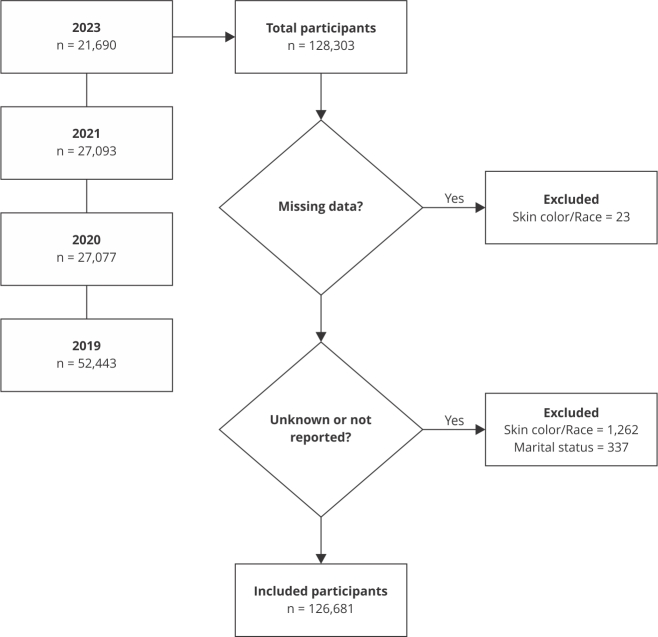
Flowchart of selected participants.

Source: Vigitel survey - 2019 ^16^, 2020 ^15^, 2021 ^14^, 2023 ^13^.

This is a population-based, repeated cross-sectional analysis using data from the *Surveillance of Risk and Protective Factors for Chronic Diseases by Telephone Survey* (Vigitel), collected in Brazil between 2019 and 2023 (n = 128,303 adults) ^13^ ^,^ ^14^ ^,^ ^15^ ^,^ ^16^. In 2019, 52,443 individuals were interviewed, followed by 27,077 in 2020, 27,093 in 2021, and 21,690 in 2023. Of the total sample (128,303), 23 individuals with missing data on skin color/race, 1,262 who answered “do not know” or were unable to report their skin color/race, and 337 who did not report their marital status were excluded. Therefore, data from 126,681 individuals were analyzed (Figure 1).

Vigitel is a nationwide telephone survey that monitors the prevalence of risk and protective factors for noncommunicable diseases (NCDs) among adults aged 18 years or older in all 26 Brazilian state capitals and the Federal District ^16^. Vigitel sampling is conducted in two stages. First, a random sample of telephone lines is selected from the Brazilian National Telecommunications Agency (Anatel, acronym in Portuguese) database and divided into city-specific subsamples. Second, one adult from each selected household (landline) or mobile phone owner is randomly selected to participate in the interview ^13^ ^,^ ^14^ ^,^ ^15^ ^,^ ^16^ ^,^ ^17^. Weighting is performed using the Rake method, based on data from the national census and official projections ^13^.

The system establishes a minimum sample size of approximately 2,000 interviews per locality (around 54,000 interviews per year) to estimate the prevalence of any factor in the adult population with a 95% confidence interval (95%CI) and a maximum 2% margin of error. In 2020 and 2021, the minimum sample size was reduced to 1,000 individuals per city, and interviews were conducted between January and April, unlike previous years, when data collection occurred throughout the entire year. In 2023, mobile phone interviews were also included ^14^ ^,^ ^15^ ^,^ ^16^ ^,^ ^17^. For more details about Vigitel’s sampling, weighting process, and methodological evolution refer to the official Brazilian Ministry of Health website (https://svs.aids.gov.br/download/Vigitel/).

### Outcome: lifetime ENDS use

The outcome variable was based on the following question: “Do you use electronic devices with liquid nicotine or shredded tobacco leaves (e.g., electronic cigarettes, electronic hookahs, heated tobacco products, or other electronic devices) to smoke or vape?” Marijuana use was explicitly excluded. Response options were: (1) yes, daily; (2) yes, less than daily; (3) no, but I have used it in the past; and (4) never used. For the analysis, responses were dichotomized into “yes” (categories 1-3) and “no” (category 4).

### Predictors: sociodemographic characteristics

The sociodemographic characteristics analyzed included geographic region, age group, biological sex (male or female), race/skin color, years of education, marital status, and having a private health insurance. The 26 state capitals and the Federal District were grouped into five macroregions according to Brazil’s official geographic division: North, Northeast, Central-West, Southeast, and South.

Age group was derived from the continuous age variable and categorized as 18-24, 25-34, 35-44, 45-54, 55-64, and ≥ 65 years. Skin color/race was determined by self-identification, following the classification system of the Brazilian Institute of Geography and Statistics (IBGE, acronym in Portuguese), with the categories: Yellow, White, Indigenous, Mixed-race (*Pardo*), and Black. To address missing or ambiguous responses, we recovered 7,399 observations using information from the open-ended question “*q69_ou*”, which allowed participants to specify their race or skin color when none of the predefined IBGE categories applied. These responses were reclassified into standard IBGE categories (White, Black, Mixed-race, Yellow, Indigenous) based on textual content and semantic similarity, including spelling variations and common vernacular expressions. This procedure enhanced data completeness and harmonization for subsequent analyses.

Marital status, initially recorded as single, married, cohabiting, separated/divorced, widowed, “do not know”, or “not reported”, was recoded into three categories: single/separated/divorced, married/cohabiting, and widowed. Participants who selected “do not know” or did not provide information were excluded from this variable. Educational level was categorized into three groups: 0-8 years, 9-11 years, and ≥ 12 years of completed schooling. Regarding private health insurance, respondents were classified as “yes” if they reported having one or more private health plans.

### Predictors: health behaviors

The health-related behaviors investigated included screen time, body mass index (BMI), binge drinking, smoking, physical inactivity, and comorbidities. Screen time was assessed using questions regarding hours spent watching television or using computers, laptops, tablet computers, and smartphones. Total screen time was dichotomized as “yes” for ≥ 3 hours/day and “no” otherwise.

BMI (kg/m^2^) was calculated from self-reported height and weight and categorized as underweight (≤ 18.5), normal weight (18.5-24.9), overweight (25.0-29.9), and obesity (≥ 30.0) ^18^. Binge drinking was defined as consumption of ≥ 5 alcoholic drinks on a single occasion for men and ≥ 4 drinks for women at least once in the past 30 days. A standard drink was defined as one can of beer, one glass of wine, or one shot of spirits (e.g., *cachaça*, whiskey, or other distilled beverages) ^16^.

Smoking status was defined by a positive response to the question, “Do you currently smoke?”, regardless of frequency, quantity, or duration. Physical inactivity followed Vigitel’s definition: no leisure-time physical activity in the past three months, no intense physical effort at work, no walking or cycling for commuting, and no participation in heavy housework ^16^. Chronic conditions assessed by Vigitel - specifically hypertension and diabetes - were used to construct the comorbidity variable, categorized as none, hypertension only, diabetes only, or both.

### Statistical analysis

The study population was described using absolute frequencies (n) and weighted prevalence (%) with 95%CI, according to sociodemographic characteristics and health behaviors, stratified by lifetime ENDS use. The Rao-Scott adjusted chi-squared test was applied to assess bivariate associations between categorical variables. Logistic regression models with survey weights were employed to estimate odds ratios (ORs) and 95%CI. Predictors were selected based on previous studies ^2^ ^,^ ^3^ ^,^ ^4^. Initially, crude models were estimated for each predictor, followed by multivariable models including all variables. All analyses were weighted to account for the complex sampling design and stratified by survey year (2019, 2020, 2021, and 2023). Statistical analyses were performed using R programming language, version 4.5.0 (http://www.r-project.org), with the *survey* package ^19^, which accommodates complex survey designs and enables extrapolation of findings to the Brazilian population. A 5% significance level was adopted.

### Ethical approval

Ethical approval was obtained from the Brazilian National Ethics Commission of the Brazilian Ministry of Health (CAAE 65610017.1.0000.0008). Vigitel databases are publicly available (http://svs.aids.gov.br/download/Vigitel/).

## Results

Between 2019 and 2023, lifetime ENDS use in Brazil increased from 6.7% in 2019 to 7.3% in 2020, remaining stable at 7.3% in 2021, and then decreased to 7% in 2023 (p < 0.001). The highest prevalence was observed in the Central-West (10.4%-13.2%) and South (7.9%-9.3%), while the Northeast consistently reported the lowest rates (3.8%-5.7%). Young adults aged 18-24 years reported the highest prevalence (17.4%-20.7%), followed by those aged 25-34 years (10.7%-13.4%), with a clear decline across older age groups. Men consistently exhibited higher prevalence (9.4%-10%), as did individuals with ≥ 12 years of education (8.5%-9.6%). When analyzed by skin color/race, the prevalence of lifetime ENDS use varied across survey years, with the highest rates observed among White individuals (7.5%) in 2019, Yellow individuals (9%) in 2020, Black individuals (10.4%) in 2021, and Indigenous individuals (16.8%) in 2023. Single, separated, or divorced participants also reported greater use (10.2%-11.9%) (Table 1).

**Table 1:** Weighted prevalence of lifetime electronic nicotine delivery systems (ENDS) use according to sociodemographic characteristics and health behaviors, stratified by survey year. *Surveillance of Risk and Protective Factors for Chronic Diseases by Telephone Survey* (Vigitel), 2019-2023.

Variables	2019 (n = 51,817)			2020 (n = 26,782)			2021 (n = 26,665)			2023 (n = 21,417)		
	n	%	95%CI	n	%	95%CI	n	%	95%CI	n	%	95%CI
Lifetime ENDS use *	1,780	6.7	6.2-7.3	863	7.3	6.3-8.2	1,043	7.3	6.4-8.2	997	7.0	6.2-7.8
Brazilian region **												
Northeast	461	4.0	3.5-4.6	195	3.8	3.0-4.6	278	5.7	4.7-6.8	233	4.3	3.3-5.2
North	393	4.9	4.1-5.7	219	4.7	3.7-5.6	260	5.5	4.3-6.6	227	4.9	3.7-6.0
Central-West	408	11.6	10.0-13.2	187	13.2	10.7-15.7	222	12.5	10.3-14.7	207	10.4	8.4-12.5
Southeast	267	7.2	6.0-8.3	135	8.2	6.2-10.1	145	6.9	5.2-8.7	154	7.8	6.1-9.5
South	251	8.1	6.7-9.4	127	7.9	6.1-9.7	138	9.3	7.1-11.5	176	9.2	7.5-10.9
Age group (years) **												
18-24	629	19.4	17.1-21.7	323	20.7	17.3-24.0	273	17.4	13.4-20.9	315	18.6	15.1-22.1
25-34	428	10.7	9.1-12.3	229	12.1	9.1-15.0	270	12.6	10.0-15.3	357	13.4	11.0-15.9
35-44	212	3.4	2.6-4.1	132	5.0	3.4-6.5	150	5.4	3.7-7.1	171	3.6	2.7-4.5
45-54	132	2.1	1.4-2.8	70	1.7	1.0-2.3	73	2.1	1.4-2.8	72	1.9	1.0-2.7
55-64	179	1.6	1.2-2.1	59	0.9	0.5-1.4	101	2.1	1.3-2.8	45	1.3	0.4-2.1
≥ 65	200	1.4	0.9-1.8	50	0.9	0.3-1.4	176	1.6	1.2-2.1	37	1.0	0.3-1.7
Sex **												
Male	1,005	9.4	8.4-10.4	494	9.8	8.2-11.4	541	10.0	8.4-11.6	568	9.7	8.2-11.1
Female	775	4.5	3.9-5.1	369	5.1	4.0-6.2	502	5.0	4.1-6.0	429	4.7	3.9-5.6
Skin color/Race ***												
Yellow	20	5.0	2.2-7.9	10	9.0	0.6-17.4	11	7.0	0.1-14.2	13	6.6	0.1-13.0
White	815	7.5	6.5-8.4	397	8.5	6.8-10.2	471	7.3	5.9-8.6	439	8.9	7.3-10.5
Indigenous	21	6.4	0.9-11.9	15	3.8	0.8-6.8	11	8.9	1.8-16.0	13	16.8	2.1-31.6
Mixed-race	751	6.2	5.4-7.1	363	6.9	5.5-8.3	428	6.5	5.3-7.7	431	5.5	4.6-6.4
Black	173	6.2	4.7-7.8	78	4.7	2.8-6.4	122	10.4	6.9-13.4	101	6.1	4.1-8.2
Marital status **												
Single/Separated/Divorced	1,232	11.0	9.9-12.0	620	11.9	10.2-13.6	673	10.9	9.4-12.4	655	10.2	8.8-11.6
Married/Cohabiting	479	2.9	2.4-3.4	219	3.0	2.1-3.9	304	3.4	2.5-4.3	325	4.2	3.3-5.1
Widowed	69	0.7	0.4-1.0	24	0.5	0.1-0.8	66	2.8	1.0-4.7	17	1.2	0.2-2.1
Years of education **												
0-8	242	2.7	2.0-3.5	97	4.4	2.4-6.4	153	2.5	1.8-3.3	108	2.5	1.6-3.4
9-11	661	7.9	6.8-8.9	345	8.2	6.7-9.7	395	8.5	6.7-10.1	439	8.3	6.9-9.8
≥ 12	877	8.9	7.8-10.0	421	8.5	6.8-10.1	495	9.6	7.9-11.4	450	8.9	7.4-10.5
Health insurance *												
No	841	6.5	5.7-7.3	386	6.5	5.2-7.8	522	6.8	5.7-7.9	563	6.6	5.5-7.8
Yes	939	7.0	6.2-7.8	477	8.1	6.7-9.6	521	8.0	6.5-9.4	434	7.6	6.3-8.9
Screen time **												
No	463	3.6	2.9-4.2	164	3.6	2.4-4.8	296	4.9	3.6-6.2	220	3.6	2.8-4.5
Yes	1,317	8.6	7.8-9.4	699	9.0	7.7-10.3	747	8.6	7.4-9.7	777	8.7	7.5-9.8
BMI ^#^												
Underweight	56	5.7	3.1-8.3	32	8.2	3.4-12.9	42	10.1	3.5-16.7	25	6.1	1.9-10.3
Normal weight	860	8.4	7.4-9.4	393	8.4	6.7-10.0	458	8.2	6.8-9.7	402	8.9	7.2-10.7
Overweight	570	5.9	5.0-6.8	278	5.9	4.6-7.2	333	6.2	4.8-7.6	357	6.1	5.0-7.3
Obesity	294	4.9	3.8-5.9	160	7.3	5.0-9.7	210	7.1	5.1-9.1	213	5.6	4.4-6.9
Binge drinking **												
No	1,040	4.3	3.8-4.8	457	4.5	3.7-5.3	655	4.9	4.1-5.7	517	4.4	3.7-5.1
Yes	740	17.2	15.2-19.2	406	17.8	14.5-21.1	388	18.1	14.8-21.4	480	17.0	14.2-19.8
Smoking **												
No	1,362	5.5	5.0-6.1	638	5.4	4.6-6.3	788	6.0	5.1-6.9	742	6.0	5.1-6.8
Yes	418	18.0	15.1-20.9	225	24.6	18.9-30.2	255	20.3	16.3-24.2	255	17.3	13.7-20.9
Physical inactivity *												
No	1,527	6.8	6.1-7.4	734	7.3	6.3-8.3	906	7.7	6.7-8.7	896	7.1	6.2-8.0
Yes	253	6.6	5.0-8.3	129	7.2	6.3-8.3	137	5.2	2.9-7.5	101	6.5	4.1-8.8
Comorbidities **												
None	1,441	8.1	7.4-8.8	727	9.0	7.8-10.3	785	8.9	7.7-10.1	845	8.6	7.6-9.7
Hypertension	248	3.4	2.4-4.4	99	3.1	1.5-4.3	178	3.9	2.3-5.2	114	3.7	2.4-5.0
Diabetes	34	3.1	1.4-4.9	18	4.4	0.8-9.7	27	4.4	1.9-6.9	16	3.0	0.0-5.9
Hypertension and diabetes	57	1.4	0.7-2.0	19	0.4	0.1-0.7	53	1.4	0.7-2.0	22	2.7	0.0-6.0

95%CI: 95% confidence interval; BMI: body mass index.

Note: variables were analyzed using the Rao-Scott adjusted chi-squared test: * non-significant for all years; ** p-value < 0.001 for all years; *** p-value < 0.001 in 2020 and 2023; ^#^ p-value < 0.001 in 2019 and 2023.

Conventional smokers (17.3%-24.6%) and binge drinkers (17%-18.1%) reported substantially higher prevalence of lifetime ENDS use compared to their counterparts. Individuals with ≥ 3 hours of daily screen time also showed higher use (8.6%-9%). Participants without comorbidities had higher prevalence (8.1%-9%) than those with diabetes (3%-4.4%), hypertension (3.1%-3.9%), or both conditions (0.4%-2.7%). By BMI, individuals with normal weight (8.2%-8.9%) and underweight (5.7%-10.1%) exhibited slightly higher prevalence than those with overweight (5.9%-6.2%) or obesity (4.9%-7.3%). Although health insurance status and physical inactivity varied across groups and over time, these differences were not statistically significant (Table 1).

In the crude models, key correlates of increased ENDS use included residence in the Central-West (OR = 2.35-3.83) or South (OR = 1.69-2.27) versus the Northeast; age 18-24 years (OR = 12.83-29.49) versus ≥ 65 years; male sex (OR = 2.03-2.22); being single, separated, or divorced (OR = 2.57-4.35) versus married/cohabiting; ≥ 12 years of schooling (OR = 2.02-4.08) versus 0-8 years; ≥ 3 hours of daily screen time (OR = 1.82-2.68); binge drinking (OR = 4.29-4.63); conventional smoking (OR = 3.28-5.66); and absence of comorbidities (OR = 6.36-23.51) compared with both hypertension and diabetes (Table 2).

**Table 2:** Crude logistic regression models examining factors associated with lifetime electronic nicotine delivery systems (ENDS) use, stratified by year. *Surveillance of Risk and Protective Factors for Chronic Diseases by Telephone Survey* (Vigitel), 2019-2023.

Variables	2019		2020		2021		2023	
	OR	95%CI	OR	95%CI	OR	95%CI	OR	95%CI
Brazilian region								
Northeast	Reference		Reference		Reference		Reference	
North	1.23	0.98-1.54	1.23	0.91-1.67	0.95	0.71-1.28	1.14	0.82-1.59
Central-West	3.14	2.55-3.88	3.83	2.83-5.20	2.35	1.78-3.11	2.62	1.91-3.58
Southwest	1.85	1.48-2.30	2.24	1.60-3.13	1.22	0.88-1.71	1.89	1.37-2.61
South	2.09	1.67-2.63	2.17	1.57-2.99	1.69	1.23-2.34	2.27	1.67-3.08
Age group (years)								
18-24	17.50	12.24-25.03	29.49	15.37-56.60	12.83	8.86-18.57	21.73	10.66-44.30
25-34	8.67	6.01-12.51	15.58	7.91-30.70	8.79	6.08-12.71	14.70	7.25-29.81
35-44	2.53	1.70-3.75	5.91	2.94-11.87	3.48	2.25-5.39	3.54	1.72-7.29
45-54	1.59	0.99-2.54	1.91	0.90-4.04	1.28	0.82-2.01	1.80	0.80-4.05
55-64	1.19	0.77-1.84	1.08	0.50-2.34	1.29	0.80-2.08	1.21	0.47-3.12
≥ 65	Reference		Reference		Reference		Reference	
Sex								
Female	Reference		Reference		Reference		Reference	
Male	2.22	1.84-2.67	2.03	1.51-2.73	2.10	1.61-2.74	2.15	1.66-2.78
Skin color/Race								
Mixed-race	Reference		Reference		Reference		Reference	
Yellow	0.80	0.43-1.49	1.34	0.47-3.81	1.09	0.35-3.35	1.20	0.41-3.51
White	1.22	1.00-1.48	1.26	0.92-1.71	1.13	0.85-1.50	1.67	1.28-2.18
Indigenous	1.03	0.41-2.60	0.53	0.23-1.25	1.41	0.57-3.46	3.47	1.19-10.13
Black	1.00	0.74-1.36	0.65	0.41-1.03	1.62	1.08-2.44	1.12	0.75-1.67
Marital status								
Single/Separated/Divorced	4.11	3.31-5.11	4.35	3.07-6.17	3.47	2.55-4.73	2.57	1.95-3.39
Married/Cohabiting	Reference		Reference		Reference		Reference	
Widowed	0.25	0.16-0.39	0.16	0.07-0.34	0.83	0.40-1.72	0.27	0.12-0.64
Years of education								
0-8	Reference		Reference		Reference		Reference	
9-11	3.02	2.22-4.12	1.96	1.17-3.28	3.56	2.47-5.13	3.54	2.35-5.32
≥ 12	3.46	2.55-4.70	2.02	1.20-3.39	4.08	2.82-5.88	3.82	2.53-5.75
Health insurance								
No	Reference		Reference		Reference		Reference	
Yes	1.08	0.90-1.30	1.27	0.95-1.70	1.19	0.91-1.55	1.16	0.90-1.50
Screen time (≥ 3 hours/day)								
No	Reference		Reference		Reference		Reference	
Yes	2.54	2.05-3.15	2.68	1.83-3.90	1.82	1.32-2.49	2.51	1.88-3.35
BMI								
Underweight	0.66	0.40-1.08	0.97	0.50-1.90	1.25	0.59-2.65	0.66	0.31-1.41
Normal	Reference		Reference		Reference		Reference	
Overweight	0.68	0.55-0.84	0.69	0.50-0.95	0.74	0.54-1.00	0.66	0.50-0.89
Obesity	0.56	0.43-0.72	0.86	0.57-1.30	0.85	0.60-1.23	0.61	0.44-0.84
Binge drinking								
No	Reference		Reference		Reference		Reference	
Yes	4.63	3.84-5.59	4.61	3.45-6.18	4.29	3.26-5.65	4.46	3.45-5.78
Smoking								
No	Reference		Reference		Reference		Reference	
Yes	3.76	3.01-4.69	5.66	4.01-7.99	3.96	2.96-5.30	3.28	2.45-4.40
Physical inactivity								
No	Reference		Reference		Reference		Reference	
Yes	0.98	0.74-1.30	0.99	0.62-1.59	0.66	0.41-1.07	0.90	0.60-1.36
Comorbidities								
None	6.36	3.94-10.25	23.51	11.75-47.02	7.14	4.24-12.03	3.38	0.98-11.71
Hypertension	2.55	1.46-4.44	7.46	3.19-17.41	2.94	1.60-5.41	1.38	0.38-4.99
Diabetes	2.31	1.10-4.87	10.96	2.65-45.33	3.36	1.56-7.26	1.09	0.22-5.43
Hypertension and diabetes	Reference		Reference		Reference		Reference	

95%CI: 95% confidence interval; BMI: body mass index; OR: odds ratio.

Adjusted logistic regression models stratified by year revealed consistent and statistically significant associations. Compared to residents of the Northeast, those living in the Central-West and South had persistently higher odds of ENDS use. The Central-West showed the strongest associations, with ORs ranging from 2.30 (95%CI: 1.68-3.15) in 2021 to 4.27 (95%CI: 3.01-6.07) in 2020. The South also showed significantly elevated odds, including an OR of 2.16 (95%CI: 1.65-2.81) in 2019 (Table 3).

**Table 3:** Adjusted logistic regression models examining factors associated with lifetime electronic nicotine delivery systems (ENDS) use, stratified by year. *Surveillance of Risk and Protective Factors for Chronic Diseases by Telephone Survey* (Vigitel), 2019-2023.

Variables	2019		2020		2021		2023	
	OR	95%CI	OR	95%CI	OR	95%CI	OR	95%CI
Brazilian region								
Northeast	Reference		Reference		Reference		Reference	
North	1.16	0.91-1.47	1.13	0.80-1.60	0.95	0.69-1.32	1.13	0.79-1.61
Central-West	3.23	2.52-4.14	4.27	3.01-6.07	2.30	1.68-3.15	2.57	1.80-3.68
Southeast	1.92	1.51-2.44	2.50	1.76-3.55	1.41	0.97-2.05	1.98	1.40-2.80
South	2.16	1.65-2.81	2.07	1.37-3.13	1.97	1.33-2.92	2.07	1.41-3.06
Age group (years)								
18-24	7.71	5.02-11.85	16.59	6.97-39.50	7.06	3.72-13.40	20.13	7.30-55.48
25-34	3.86	2.50-5.96	6.82	2.92-15.95	4.55	2.49-8.31	12.29	4.51-33.53
35-44	1.30	0.84-2.01	3.02	1.31-6.96	1.90	1.06-3.43	2.75	1.05-7.21
45-54	0.96	0.58-1.59	1.05	0.45-2.43	0.80	0.45-1.44	1.57	0.58-4.24
55-64	0.80	0.51-1.26	0.60	0.26-1.42	0.95	0.52-1.73	1.14	0.40-3.20
≥ 65	Reference		Reference		Reference		Reference	
Sex								
Female	Reference		Reference		Reference		Reference	
Male	1.65	1.35-2.03	1.66	1.22-2.25	1.66	1.24-2.24	1.81	1.38-2.39
Skin color/Race								
Mixed-race	Reference		Reference		Reference		Reference	
Yellow	1.06	0.57-1.98	1.75	0.53-5.75	1.52	0.42-5.59	1.38	0.57-3.32
White	1.13	0.90-1.41	1.08	0.78-1.49	0.99	0.71-1.38	1.47	1.09-2.00
Indigenous	1.26	0.45-3.57	0.55	0.24-1.26	1.79	0.63-5.05	3.55	1.25-10.07
Black	0.72	0.52-1.00	0.51	0.31-0.86	1.28	0.83-1.98	0.82	0.52-1.28
Marital status								
Single/Separated/Divorced	1.59	1.23-2.06	1.65	1.05-2.58	1.51	1.04-2.19	1.13	0.83-1.55
Married/Cohabiting	Reference		Reference		Reference		Reference	
Widowed	0.54	0.33-0.90	0.61	0.24-1.53	2.62	1.13-6.09	1.05	0.41-2.70
Years of education								
0-8	Reference		Reference		Reference		Reference	
9-11	1.33	0.93-1.91	0.74	0.45-1.24	2.00	1.30-3.08	1.67	1.05-2.64
≥ 12	1.47	1.02-2.12	0.75	0.45-1.26	2.18	1.40-3.39	1.89	1.15-3.12
Health insurance								
No	Reference		Reference		Reference		Reference	
Yes	1.02	0.83-1.26	1.41	1.06-1.88	1.11	0.83-1.49	1.07	0.79-1.44
Screen time (≥ 3 hours/day)								
No	Reference		Reference		Reference		Reference	
Yes	1.40	1.11-1.78	1.42	0.98-2.04	1.12	0.80-1.58	1.40	1.03-1.89
BMI								
Underweight	0.63	0.37-1.09	0.92	0.45-1.89	1.26	0.58-2.74	0.54	0.22-1.31
Normal	Reference		Reference		Reference		Reference	
Overweight	0.94	0.75-1.20	1.10	0.78-1.54	0.99	0.70-1.39	0.76	0.55-1.07
Obesity	0.88	0.65-1.18	1.86	1.20-2.89	1.41	0.94-2.12	0.78	0.54-1.12
Binge drinking								
No	Reference		Reference		Reference		Reference	
Yes	2.58	2.08-3.18	2.49	1.81-3.42	2.78	2.02-3.84	2.86	2.13-3.85
Smoking								
No	Reference		Reference		Reference		Reference	
Yes	3.38	2.60-4.40	5.79	3.95-8.48	4.38	3.02-6.37	2.92	2.07-4.12
Physical inactivity								
No	Reference		Reference		Reference		Reference	
Yes	1.18	0.87-1.61	0.98	0.62-1.56	0.85	0.51-1.40	1.27	0.78-2.05
Comorbidities								
None	1.41	0.77-2.56	5.01	2.19-11.49	1.69	0.93-3.09	0.47	0.15-1.44
Hypertension	1.55	0.81-2.97	3.70	1.53-8.97	1.52	0.79-2.93	0.55	0.19-1.63
Diabetes	1.54	0.71-3.33	3.37	0.70-16.26	1.96	0.81-4.75	0.51	0.12-2.17
Hypertension and diabetes	Reference		Reference		Reference		Reference	

95%CI: 95% confidence interval; BMI: body mass index; OR: odds ratio.

A clear age gradient was observed, with young adults aged 18-24 years consistently exhibiting the highest odds compared to those aged ≥ 65 years, ranging from OR = 7.06 (95%CI: 3.72-13.40) in 2021 to OR = 20.13 (95%CI: 7.30-55.48) in 2023. Individuals aged 25-34 years also showed elevated odds across all years. Men had higher odds than women in every year (OR = 1.65-1.81). Compared to Mixed-race individuals, White participants had significantly higher odds in 2023 (OR = 1.47; 95%CI: 1.09-2.00), as did Indigenous participants (OR = 3.55, 95%CI: 1.25-10.07), while other groups showed inconsistent associations.

Being single, separated, or divorced was significantly associated with higher odds of lifetime ENDS use from 2019 to 2021, though this effect weakened and was no longer significant in 2023. Higher educational attainment (≥ 12 years) was significantly associated with increased odds in 2021 (OR = 2.18; 95%CI: 1.40-3.39) and 2023 (OR = 1.89; 95%CI: 1.15-3.12). Possession of private health insurance was associated with higher odds only in 2020 (OR = 1.41; 95%CI: 1.06-1.88). Daily screen time of ≥ 3 hours was significant in 2019 (OR = 1.40; 95%CI: 1.11-1.78) and 2023 (OR = 1.40; 95%CI: 1.03-1.89).

BMI showed no consistent associations with ENDS use over the analyzed years, although obesity was linked to higher odds in 2020 (OR = 1.86; 95%CI: 1.20-2.89). Binge drinking was consistently and strongly associated across all years, with ORs ranging from 2.49 (95%CI: 1.81-3.42) in 2020 to 2.86 (95%CI: 2.13-3.85) in 2023. Conventional smoking also emerged as one of the strongest predictors, especially in 2020 (OR = 5.79; 95%CI: 3.95-8.48) and 2021 (OR = 4.38; 95%CI: 3.02-6.37). Physical inactivity showed no significant associations. Notably, absence of comorbidities was strongly associated with ENDS use in 2020 (OR = 5.01; 95%CI: 2.19-11.49), suggesting that users tend to be younger and healthier.

Overall, younger individuals, males, residents of the Central-West and South, those with higher educational attainment, individuals reporting binge drinking, conventional smokers, and those with longer daily screen exposure consistently demonstrated higher odds of lifetime ENDS use. In contrast, no consistent associations were observed for physical inactivity, BMI, or comorbidities, although lack of chronic conditions was linked to higher odds in earlier years.

## Discussion

This study identifies a consistent sociodemographic and behavioral profile of lifetime ENDS users in Brazil, alongside a rising prevalence from 2019 to 2023. ENDS use was strongly associated with younger age, male sex, higher educational attainment, residence in the Central-West and South, binge drinking, and current smoking. These findings indicate that lifetime ENDS use is concentrated among younger, healthier, and more socioeconomically advantaged groups. However, its association with other risk factors - such as living alone, excessive screen time, and racial disparities, particularly among White and Indigenous populations - underscores the complex interplay of social and behavioral determinants.

Conventional cigarettes in Brazil are among the most affordable worldwide, which facilitates their accessibility and consumption ^9^. In contrast, ENDS are significantly more expensive, limiting their consumption to groups with greater purchasing power ^20^. This cost disparity has led to a concentration of lifetime ENDS use among younger individuals with higher educational and income levels, who often perceive these products as a modern alternative and, mistakenly, as less harmful than traditional cigarettes.

The higher prevalence observed in the Central-West and South compared with the Northeast may reflect differences in access, enforcement, and cultural influences ^10^. The high cost of ENDS suggests that their use is concentrated among wealthier youth. Considering Brazil’s regional socioeconomic disparities, it is reasonable to expect higher prevalence rates in the Central-West, South, and Southeast compared to the Northeast, which presents lower per capita income and Human Development Index (HDI) levels ^2^. Two Brazilian studies support this regional pattern, emphasizing the need for context-specific policies ^10^ ^,^ ^20^. This highlights the importance of tailoring ENDS control policies to regional contexts ^21^.

Age was a strong predictor, with young adults (18-24 years) showing markedly higher odds compared to older adults, with increases ranging from 606% to 1,013%. This aligns with global findings and reinforces the need for youth-focused interventions ^22^ ^,^ ^23^ ^,^ ^24^. Adolescents and young adults seem particularly susceptible due to the appeal of flavored products, strong exposure to digital marketing, and the illegal commercialization of ENDS in social environments such as parties and events ^2^ ^,^ ^8^ ^,^ ^10^. Additionally, insufficient regulation of online marketing practices creates favorable conditions for manufacturers.

The higher prevalence among men mirrors traditional smoking patterns and may reflect social norms and risk-taking behaviors ^25^ ^,^ ^26^. Several studies corroborate this finding ^3^ ^,^ ^10^ ^,^ ^20^ ^,^ ^23^ ^,^ ^24^ ^,^ ^27^, reinforcing the importance of gender-sensitive strategies.

Skin color/race disparities were also evident. Variations in the racial profile of ENDS users suggests a progressive social diffusion of these products in Brazil. Initially concentrated among White individuals with higher purchasing power (2019-2020), ENDS use has expanded to racialized groups, particularly Black and Indigenous populations (2021-2023). Such shift is possibly due to decreasing costs, growth of informal markets, and wider circulation via social media and youth spaces. This shift reflects economic, structural, and territorial inequalities that shape exposure to health risks and access to consumer goods, as well as cultural changes linked to the aesthetic and identity-related appeal of vaping among young people. In addition, potential sampling and coverage effects in the Vigitel survey, especially among smaller population subgroups, may also have contributed to annual fluctuations in prevalence estimates. Overall, this pattern expresses the interplay of social determinants, cultural dynamics, and methodological limitations, revealing the complex and unequal adoption of new forms of nicotine consumption in the country. Notably, a French adolescent survey reported higher odds among Black and Brown individuals compared to White individuals ^27^, suggesting dynamic and context-specific racial patterns.

Marital status was also significant. Single, separated, or divorced individuals reported higher odds of ENDS use compared to married or cohabiting participants. Similar findings were reported in Brazil ^28^ and the United States ^29^ ^,^ ^30^, although other U.S. data showed higher prevalence among widowed and divorced individuals ^21^. In our results, lower odds among widowed individuals in 2019, followed by higher odds in 2021, may reflect grief and psychosocial vulnerabilities linked to COVID-19-related losses ^31^ ^,^ ^32^.

Higher education was associated with greater ENDS use in 2019, 2021, and 2023, possibly reflecting higher income, greater access, increased experimentation, and perceptions of ENDS as modern or less harmful ^22^ ^,^ ^33^. Similar findings have been reported in China ^34^ ^,^ ^35^ and Brazil ^10^. Higher education may also be linked to greater screen time, potentially increasing exposure to online ENDS marketing, consistent with our findings.

Daily screen time ≥ 3 hours was associated with ENDS use, with 40% higher odds in 2019 and 2023, likely reflecting greater exposure to online promotion, especially among youth ^36^. Regulating the internet and digital media is essential to curb the growing advertising of electronic cigarettes in Brazil, especially considering Anvisa has banned the commercialization, importation, and advertising of these products since 2009 ^9^. Despite the ban, companies and digital media influencers exploit loopholes on social media and digital platforms to promote these devices covertly, often targeting young audiences. Without effective oversight and clear rules for the digital environment, these practices often go unpunished, undermining public health and weakening the authority of existing legislation. Therefore, it is urgent to strengthen regulation and monitoring of online advertising to ensure that Brazilian laws are also enforced in the virtual space ^2^ ^,^ ^9^ ^,^ ^36^. The lack of significant associations in 2020 and 2021 may reflect pandemic-related behavioral changes. Continued monitoring is essential.

Risk behaviors were strongly associated with ENDS use. Tobacco smoking (up to 479% higher odds) and binge drinking (up to 176% higher odds) indicate behavioral clustering, consistent with previous studies ^26^ ^,^ ^32^ ^,^ ^37^ ^,^ ^38^ ^,^ ^39^ ^,^ ^40^ ^,^ ^41^. Although more prevalent among smokers, ENDS use also occurs among young, educated, and higher-income individuals, suggesting potential initiation by these devices and reinforcing the need for integrated prevention strategies.

ENDS use among young people is particularly concerning due to its negative health impacts, including damage to the respiratory system, increased cardiovascular risk, and impaired neurological development ^3^ ^,^ ^4^. Dual use with conventional cigarettes further amplifies these harms and hinders nicotine cessation. Studies show that young dual users tend to exhibit greater dependence and share several risk factors such as higher exposure to tobacco advertising, emotional vulnerabilities, and lower risk perception, making cessation more difficult ^2^ ^,^ ^20^ ^,^ ^21^.

Although crude models indicated higher odds of ENDS use among individuals with no comorbidities, hypertension, nor diabetes, these associations were attenuated after adjustment, remaining statistically significant only for individuals without comorbidities or with isolated hypertension in 2020. Notably, It is noteworthy that the reference category consisted of individuals with both comorbidities (hypertension and diabetes), who are typically older and may avoid additional risk behaviors ^40^. BMI, physical inactivity, and health insurance were not significantly associated after adjustment, suggesting limited relevance in this context.

These findings have important public health implications. The clustering of ENDS use with alcohol and tobacco highlights the need for integrated youth-focused prevention ^20^. Regional and sociodemographic disparities reinforce the importance of targeted, context-specific interventions ^27^. Combined strategies involving education, digital media regulation, and enforcement are essential to reduce ENDS use and its health impacts ^30^ ^,^ ^41^.

Strengths of this study include the use of large, nationally representative, multi-year data, enabling robust analyses and subgroup comparisons. The inclusion of sociodemographic, behavioral, and health-related variables strengthens the multidimensional risk profile. However, limitations include the cross-sectional design, self-reported measures, potential underrepresentation of specific groups, and limited data on ENDS type, duration, and frequency. Differences in sampling between 2020 and 2021 (due to earlier data collection) and the inclusion of mobile phones in 2023 may have affected comparability. Despite these limitations, the study provides critical insights to guide prevention and policy development.

## Conclusion

This study highlights increasing ENDS use in Brazil, particularly among young adults, men, individuals with higher educational attainment, and those engaging in other risk behaviors. These findings underscore the urgent need to strengthen enforcement at social events and regulate online sales and marketing, considering regional disparities. Preventive strategies should prioritize education, regulation, and media literacy, especially among youth. Continued surveillance is essential to inform and strengthen evidence-based interventions.

## Data Availability

The databases used in the study, including extraction codes, analyses, and results, are available in the repository: (https://github.com/greenmadox/ENDS_VIGITEL).
